# Impact of *XRCC2* Arg188His Polymorphism on Cancer Susceptibility: A Meta-Analysis

**DOI:** 10.1371/journal.pone.0091202

**Published:** 2014-03-12

**Authors:** Yazhou He, Yuanchuan Zhang, Chengwu Jin, Xiangbing Deng, Mingtian Wei, Qingbin Wu, Tinghan Yang, Yanhong Zhou, Ziqiang Wang

**Affiliations:** 1 Department of Gastrointestinal Surgery, West China Hospital, Sichuan University, Chengdu, Sichuan Province, P.R China; 2 West China School of Medicine/West China Hospital, Sichuan University, Chengdu, Sichuan Province, P.R China; 3 Department of Laboratory Medicine, West China Hospital, Sichuan University, Chengdu,Sichuan Province, P.R China; Shanghai Jiao Tong University School of Medicine, China

## Abstract

**Background:**

Association between the single nucleotide polymorphism rs3218536 (known as Arg188His) located in the X-ray repair cross complementing group 2 (*XRCC2*) gene and cancer susceptibility has been widely investigated. However, results thus far have remained controversial. A meta-analysis was performed to identify the impact of this polymorphism on cancer susceptibility.

**Methods:**

PubMed and Embase databases were searched systematically until September 7, 2013 to obtain all the records evaluating the association between the *XRCC2* Arg188His polymorphism and the risk of all types of cancers. We used the odds ratio (OR) as measure of effect, and pooled the data in a Mantel-Haenszel weighed random-effects meta-analysis to provide a summary estimate of the impact of this polymorphism on breast cancer, ovarian cancer and other cancers. All the analyses were carried out in STATA 12.0.

**Results:**

With 30868 cases and 38656 controls, a total of 45 case-control studies from 26 publications were eventually included in our meta-analysis. No significant association was observed between the *XRCC2* Arg188His polymorphism and breast cancer susceptibility (dominant model: OR = 0.94, 95%CI = 0.86–1.04, P = 0.232). However, a significant impact of this polymorphism was detected on decreased ovarian cancer risk (dominant model: OR = 0.83, 95%CI = 0.73–0.95, P = 0.007). In addition, we found this polymorphism was associated with increased upper aerodigestive tract (UADT) cancer susceptibility (dominant model: OR = 1.51, 95%CI = 1.04–2.20, P = 0.032).

**Conclusion:**

The Arg188His polymorphism might play different roles in carcinogenesis of various cancer types. Current evidence did not suggest that this polymorphism was directly associated with breast cancer susceptibility. However, this polymorphism might contribute to decreased gynecological cancer risk and increased UADT cancer risk. More preclinical and epidemiological studies were still imperative for further evaluation.

## Introduction

As the core component of cell nucleus, DNA suffers from various damaging agents such as chemicals, radiations and some endogenous elements. Under these damages, single strand breaks (SSBs) occur. Subsequently, unrepaired SSBs lead to double strand breaks (DSBs) during the S phase of cell cycle [1]. It has been demonstrated that accumulation of unrepaired DSBs can cause cell death and initiate malignancies [2], which highlights the disorder of DNA repair as the key role in tumorigenesis.

There are several mechanisms repairing DSBs, among which homologous recombination repair (HRR) is the key pathway functioning in the S phase of somatic mammalian cell cycle [2]. Defective HRR has been reported to be closely related to human cancers [3]. During the HRR process, a sister chromatid is provided as a template and the homologous sequence of DNA is aligned. A wide range of crucial molecules have been identified to participate in the HRR process [4].Recently, researches have revealed that RAD51 paralogs (RAD51B, RAD51C, RAD51D, XRCC2, XRCC3) could serve as central proteins during the HRR process [5].

Coded by X-ray repair cross complementing group 2(*XRCC2*) gene, the XRCC2 protein, together with other proteins, RAD51L3 for example [6], forms a complex which plays a critical role in chromosome segregation and apoptotic response to DSBs [7]. Johnson et al. observed over 100-folds of HRR reduction in the XRCC2 deficient hamster cells compared with the parental cells [8], which confirmed the essential function of the XRCC2 protein for the HRR process. Studies have found that single nucleotide polymorphisms (SNPs) in the DNA repair gene might modify DNA repair capacity and subsequently influence susceptibility of cancer [9]. Recently, studies have focused on the influence of SNPs in the *XRCC2* gene on genomic instability and tumorigenesis. However, the exact function of SNPs in the *XRCC2* gene in response to different DNA damaging agents still remained unclear. There is a G to A polymorphism located in exon 3 of the *XRCC2* gene resulting in a substitution of histidine (His) for arginine (Arg). Known as Arg188His (R188H, rs3218536), this polymorphism has been widely investigated to explore its potential impact on cancer susceptibility.

A previous meta-analysis reported no significant association between *XRCC2* Arg188His polymorphism and breast cancer risk, whereas only one specific cancer type included led to its limitation and the unexplained heterogeneity might reduce the validity of the conclusion [10]. It was widely reported that a single SNP was related to multiple human cancers, which revealed critical common characteristics among mechanisms of different types of cancer [11], [12]. Recently, a large number of studies have attempted to identify the association between this polymorphism and other human cancers such as ovarian cancer [13], thyroid cancer [14], and colorectal cancer [15]. However, results of these studies still remain inconsistent rather than conclusive.

As to other SNPs within the *XRCC2* gene such as rs718282, rs3218373, and rs6464268, meta-analysis could not be performed due to insufficient published studies. Given the essential role of *XRCC2* gene in tumorigenesis, and a relatively small sample size for a single study, we conducted a meta-analysis including all published literature to evaluate the impact of the *XRCC2* Arg188His polymorphism on susceptibility of all available types of cancer.

## Materials and Methods

### Search strategy

A systematic search with no limits was performed in PubMed and Embase databases to identify all the studies on the association between *XRCC2* Arg188His polymorphism and cancer risk (last search updated on Sep. 7, 2013). The following search terms were adopted jointly: ‘polymorphism or variant or mutation’, ‘cancer or carcinoma’ and ‘XRCC2’. In addition, cited references of eligible studies and relevant articles were hand-searched as appropriate.

### Inclusion and exclusion criteria

Studies consistent with the following criteria were included in our meta-analysis: (1) assessing the association between the *XRCC2* Arg188His polymorphism and risk for cancer; (2) case-control studies; (3) sufficient data (a detailed number of genotypes including Arg/Arg, Arg/His and His/His in both the case and the control group); (4) English articles. Correspondingly, studies in accord with any of the following items were excluded: (1) reviews and abstracts; (2) departure from Hardy–Weinberg equilibrium (HWE) detected in controls. Studies recruiting patients during overlapping time in the same hospital were regarded as repeating data; only studies with larger numbers of patients and controls were included.

### Data extraction

According to the inclusion criteria above, two investigators (YZH and YCZ) extracted information independently from all eligible studies. Disagreements were resolved by discussion. If consensus could not be reached, we consulted a third reviewer (ZQW). Items extracted from all included studies were listed as follows: first author, year of publication, country of origin, ethnicity, cancer type, source of control groups (population-based or hospital-based), number of cases and controls, genotyping methods, selection criteria for controls ,minor allele frequency (MAF) and fitness of HWE in controls. For articles including separate case-control studies for different study centers, different ethnic groups or cancer types, data were collected separately whenever possible due to the potential between-study heterogeneity.

### Statistical analysis

Odds ratios (ORs) and corresponding 95% confidence intervals (CIs) were used as the measure of effect to evaluate the strength of association between the *XRCC2* Arg188His polymorphism and cancer susceptibility. Data were pooled using the Mantel-Haenszel method (P<0.05 was considered as statistically significant). For this polymorphism, the dominant model (Dom:His/His + Arg/His vs. Arg/Arg), recessive model (Rec:His/His vs. Arg/His +Arg/Arg) and additive model (Add: comparison of weights for His/His as ‘2’, Arg/His as ‘1’ and Arg/Arg as ‘0’) were chosen to calculate the pooled ORs.

In view of the potential heterogeneity among studies with different cancer types and various ethnicities, the random-effects model (the DerSimonian and Laird method) was adopted. Heterogeneity among eligible studies was assessed by Cochran's Q test. The P-value<0.1 indicated significant heterogeneity according to the previous study [16]. We conducted stratified analyses by variables such as cancer types, and ethnicity. Notably, if significant heterogeneity was observed after stratified analysis, a meta-regression analysis was performed to explore the potential origin of heterogeneity. By sequentially excluding every single study, we conducted sensitivity analysis to identify stability of the results and check whether any single study contributed to the heterogeneity significantly.

Considering that enough studies should be included in order to observe the variation trend of the ORs effectively, we performed cumulative meta-analysis as evidence accumulated by time when 10 or more studies were included. Hardy-Weinberg equilibrium (HWE) was assessed by Pearson's χ2 test, and P<0.05 was regarded as departure from HWE. We checked the symmetry of Begg's funnel plot and the results of Egger's test to assess the publication bias. All of the statistical analyses were conducted by STATA (version 12.0; StataCorp,College Station, Tex).

## Results

### Identification and characteristics of eligible studies

**Figure 1:**
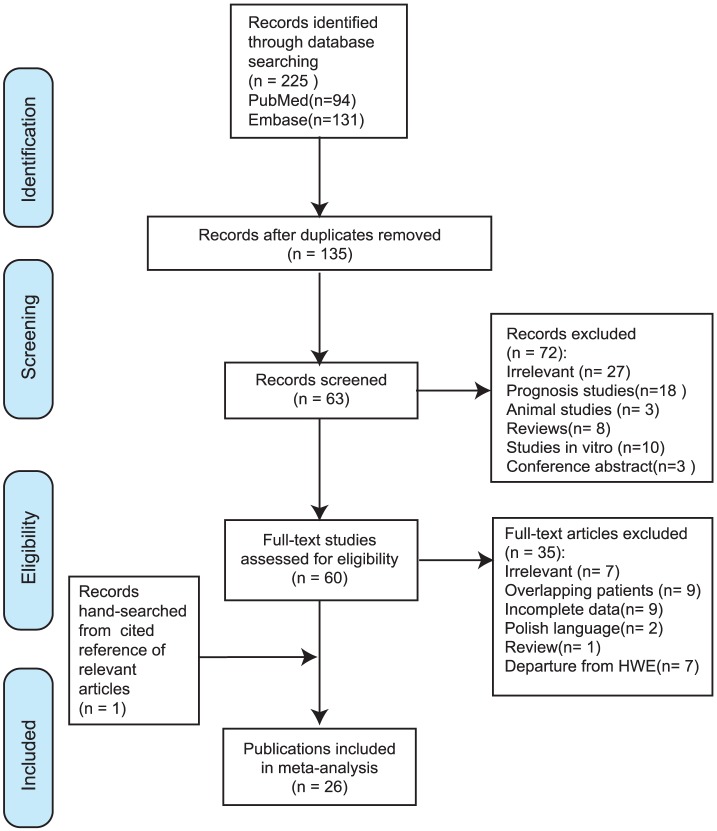
PRISMA flow diagram for study selection. After comprehensive screening, 26 publications were finally included.

After initial search with duplicates discarded, a total of 225 records of publications were yielded. Following the predefined inclusion and exclusion criteria, eventually 26 articles with 45 case-control studies were included in this meta-analysis (details in Fig.1).

The basic characteristics of eligible studies were listed in Table.1. Among these eligible studies, there were 14 case-control studies from 7 articles that investigated association between the Arg188His polymorphism and breast cancer risk [17]–[23], and 9 studies from 6 articles focused on gynecological cancer such as ovarian cancer [13], [23], [24],endometrial cancer [25], [26] and cervical cancer [27]. Moreover, a wide range of other cancer types were covered by studies including thyroid [14], [28], [29], pancreatic [30], colorectal [15], [31], bladder [32], [33], brain [34], skin [35], upper aerodigestive tract (UADT) [36], [37] and lung [38], [39] cancer. As to ethnicity, most of the eligible studies were performed in Caucasians, except for 1 study in African Americans [20] (Table 1). Furthermore, 3 studies reported association in both smokers and non-smokers [30], [32], [37]. As shown in Table 1, the source of controls was divided into hospital-based and population-based, and different selecting criteria for the control group were adopted. The distributions of genotypes for all genetic models were summarized in Table 2.

**Table 1:** Baseline characteristics of eligible case-control studies.

First Author	N*	Year	Cancer Type	Country	Ethnicity	Source of Control	Cases/Controls	Genotyping Method	Selection criteria for controls
									
Brooks [17]		2008	Breast cancer	US	Mixed	NA	602/602	PCR-RFLP	Age
García-Closas [18]		2006	Breast cancer	Poland	Caucasian	PB	1981/2280	NA	Age
Han [19]		2004	Breast cancer	US	Mixed	PB	952/1237	TaqMan/ABI PRISM	Age
Millikan [20]	1	2005	Breast cancer	US	African American	PB	765/678	TaqMan	Age/Ethnicity
Millikan [20]	2	2005	Breast cancer	US	Caucasian	PB	1268/1134	TaqMan	Age/Ethnicity
Pharoah [21]	1	2006	Breast cancer	UK	Caucasian	HB	254/194	PCR-RFLP	NA
Pharoah [21]	2	2006	Breast cancer	UK	Caucasian	PB	585/598	PCR-RFLP	NA
Pharoah [21]	3	2006	Breast cancer	UK	Caucasian	HB	863/845	TaqMan	NA
Pharoah [21]	4	2006	Breast cancer	UK	Caucasian	PB	1865/1402	TaqMan	Age
Pharoah [21]	5	2006	Breast cancer	UK	Caucasian	PB	4364/5246	TaqMan	Region
Pharoah [21]	6	2006	Breast cancer	UK	Caucasian	PB	973/968	TaqMan	NA
Pharoah [21]	7	2006	Breast cancer	UK	Mixed	HB	712/1046	TaqMan	NA
Romanowicz-Makowska [22]		2012	Breast cancer	Poland	Caucasian	NA	790/798	PCR-RFLP	NA
Webb [23]	1	2005	Breast cancer	Australia	Caucasian/Mixed	PB	1447/783	ABI PRISM	Age
Webb [23] **	2	2005	Breast cancer	Australia	Caucasian	PB	1298/658	ABI PRISM	Age
Auranen [13]	1	2005	Ovarian cancer	UK	Caucasian	PB	729/842	TaqMan/ABI PRISM	Region
Auranen [13]	2	2005	Ovarian cancer	Denmark	Caucasian	PB	269/561	TaqMan/ABI PRISM	Region
Auranen [13]	3	2005	Ovarian cancer	US	Caucasian	PB	315/404	TaqMan/ABI PRISM	Age/Ethnicity
Auranen [13]	4	2005	Ovarian cancer	UK	Caucasian	PB	275/1811	TaqMan/ABI PRISM	NA
Beesley [24]		2007	Ovarian cancer	Australia	Caucasian	PB	923/818	MALDI-TOF-MS	NA
Webb [23]	3	2005	Ovarian cancer	Australia	Caucasian/Mixed	PB	524/1118	ABI PRISM	Age
Webb [23] **	4	2005	Ovarian cancer	Australia	Caucasian	PB	430/950	ABI PRISM	Age
Han [25]		2004	Endometrial cancer	US	Mixed	NA	217/659	TaqMan/ABI PRISM	Age
Romanowicz-Makowska [26]		2012	Endometrial cancer	Poland	Caucasian	NA	230/236	PCR-RFLP	NA
Pérez [27]		2013	Cervical cancer	Argentina	Caucasian	PB	117/205	PCR EIA	NA
Rajaraman [34]	1	2010	Glioma	US	Mixed	HB	342/468	TaqMan	Age/Ethnicity/Sex/Hospital
Rajaraman [34]	2	2010	Meningioma	US	Mixed	HB	121/468	TaqMan	Age/Ethnicity/Sex/Hospital
Rajaraman [34]	3	2010	Acoustic neuroma	US	Mixed	HB	65/468	TaqMan	Age/Ethnicity/Sex/Hospital
Han [35]	1	2004	Melanoma	US	Mixed	PB	214/864	TaqMan/ABI PRISM	Age/Ethnicity
Han [35]	2	2004	Squamous cell cancer	US	Mixed	PB	284/864	TaqMan/ABI PRISM	Age/Ethnicity
Han [35]	3	2004	Basal cell cancer	US	Mixed	PB	298/864	TaqMan/ABI PRISM	Age/Ethnicity
Benhamou [36]	1	2004	Oral/Pharyngeal cancer	France	Caucasian	HB	119/165	PCR-RFLP	Age/Sex/Hospital
Benhamou [36]	2	2004	Laryngeal cancer	France	Caucasian	HB	127/165	PCR-RFLP	Age/Sex/Hospital
Romanowicz-Makowska [37]		2012	Larygeal cancer	Poland	Caucasian	NA	253/253	PCR-RFLP	NA
Jiao [30]	1	2008	Pancreatic Cancer	US	Caucasian	HB	386/418	PCR-RFLP	NA
Jiao [30]	2	2008	Pancreatic Cancer	US	Caucasian	HB	24/19	PCR-RFLP	NA
Curtin [15]		2009	Colorectal cancer	US	Mixed	PB	1209/1380	SNPlex	Age/Sex
Krupa [31]		2011	Colorectal cancer	Poland	Caucasian	HB	100/100	PCR-RFLP	Sex/Age
Figueroa [33]		2007	Bladder cancer	Spain	Caucasian	HB	1138/1129	TaqMan	Age/Sex/Ethnicity/Region
Matullo [32]		2005	Bladder cancer	Italy	Caucasian	HB	156/109	PCR-RFLP	Age/Region
Hung [38]		2008	Lung cancer	France	Mixed	HB/PB	2417/3812	TaqMan/PCR-RFLP	NA
Zienolddiny [39]		2005	Lung cancer	Norway	Caucasian	PB	312/293	APEX	Age/Smoke
Bastos [28]		2009	Thyroid cancer	Portugal	Caucasian	PB	109/217	PCR-RFLP	NA
Fayaz [29]		2012	Thyroid cancer	Iran	Caucasian	PB	50/50	PCR-HRM	NA
García-Quispes [14]		2011	Thyroid cancer	Spain	Caucasian	PB	397/477	iPLEX	NA

* Number °f case-control studies separately reported by articles.

^**^Studies included only in the subgroup meta-analysis of ethnicity.

PB: population-based; HB: hospital-based; NA: not available; PCR: polymerase chain reaction; PCR-RFLP: polymerase chain reaction-restriction fragment length polymorphism; MALDI-TOF-MS: matrix-assisted laser desorption/ionization time-of-flight mass spectrometry; PCR-HRM: polymerase chain reaction -high resolution melting; APEX: arrayed primer extension; PCR EIA: Polymerase Chain Reaction-Enzyme Immunoassay.

**Table 2:** Genotype distribution of *XRCC2* Arg188His polymorphism.

First Author	N*	Year	Cancer Type	Case				Control				HWE	MAF in controls
				N	Arg/Arg	Arg/His	His/His	N	Arg/Arg	Arg/His	His/His		
Brooks [17]		2008	Breast cancer	602	515	83	4	602	519	78	5	Yes	0.07
García-Closas [18]		2006	Breast cancer	1981	1763	212	6	2280	1983	281	16	Yes	0.07
Han [19]		2004	Breast cancer	952	811	134	7	1237	1066	165	6	Yes	0.07
Millikan [20]	1	2005	Breast cancer	765	744	21	0	678	653	25	0	Yes	0.02
Millikan [20]	2	2005	Breast cancer	1268	1084	176	8	1134	982	145	7	Yes	0.07
Pharoah [21]	1	2006	Breast cancer	254	222	31	1	194	161	32	1	Yes	0.09
Pharoah [21]	2	2006	Breast cancer	585	491	91	3	598	507	84	7	Yes	0.08
Pharoah [21]	3	2006	Breast cancer	863	695	152	16	845	698	136	11	Yes	0.09
Pharoah [21]	4	2006	Breast cancer	1865	1662	198	5	1402	1177	214	11	Yes	0.08
Pharoah [21]	5	2006	Breast cancer	4363	3698	633	32	5246	4385	824	37	Yes	0.09
Pharoah [21]	6	2006	Breast cancer	973	818	145	10	968	807	155	6	Yes	0.09
Pharoah [21]	7	2006	Breast cancer	712	587	122	3	1046	882	161	3	Yes	0.08
Romanowicz-Makowska [22]		2012	Breast cancer	790	212	374	204	798	202	406	190	Yes	0.49
Webb [23]	1	2005	Breast cancer	1447	1251	187	9	783	675	101	7	Yes	0.07
Webb [23] **	2	2005	Breast cancer	1298	1113	177	8	658	562	90	6	Yes	0.08
Auranen [13]	1	2005	Ovarian cancer	729	629	98	2	842	704	129	9	Yes	0.09
Auranen [13]	2	2005	Ovarian cancer	269	238	31	0	561	484	75	2	Yes	0.07
Auranen [13]	3	2005	Ovarian cancer	315	260	54	1	404	331	68	5	Yes	0.10
Auranen [13]	4	2005	Ovarian cancer	275	251	23	1	1811	1538	267	6	Yes	0.08
Beesley [24]		2007	Ovarian cancer	923	799	117	7	818	696	115	7	Yes	0.08
Webb [23]	3	2005	Ovarian cancer	524	451	68	5	1118	952	156	10	Yes	0.08
Webb [23] **	4	2005	Ovarian cancer	430	364	63	3	950	802	140	8	Yes	0.08
Han [25]		2004	Endometrial cancer	217	183	32	2	659	557	99	3	Yes	0.08
Romanowicz-Makowska [26]		2012	Endometrial cancer	230	61	111	58	236	57	126	53	Yes	0.49
Pérez [27]		2013	Cervical cancer	117	106	11	0	205	165	40	0	Yes	0.10
Rajaraman [34]	1	2010	Glioma	342	285	56	1	468	395	70	3	Yes	0.08
Rajaraman [34]	2	2010	Meningioma	121	106	14	1	468	395	70	3	Yes	0.08
Rajaraman [34]	3	2010	Acoustic neuroma	65	57	8	0	468	395	70	3	Yes	0.08
Han [35]	1	2004	Melanoma	214	181	31	2	864	730	127	7	Yes	0.08
Han [35]	2	2004	Squamous cell cancer	284	239	42	3	864	730	127	7	Yes	0.08
Han [35]	3	2004	Basal cell cancer	298	257	38	3	864	730	127	7	Yes	0.08
Benhamou [36]	1	2004	Oral/Pharyngeal cancer	119	92	24	3	165	142	22	1	Yes	0.07
Benhamou [36]	2	2004	Larygeal cancer	127	109	18	0	165	142	22	1	Yes	0.07
Romanowicz-Makowska [37]		2012	Larygeal cancer	253	230	22	1	253	240	13	0	Yes	0.03
Jiao [30]	1	2008	Pancreatic Cancer	386	340	44	2	418	368	49	1	Yes	0.06
Jiao [30]	2	2008	Pancreatic Cancer	24	21	3	0	19	16	3	0	Yes	0.08
Curtin [15]		2009	Colorectal cancer	1209	1014	185	10	1380	1167	204	9	Yes	0.08
Krupa [31]		2011	Colorectal cancer	100	75	18	7	100	84	14	2	Yes	0.09
Figueroa [33]		2007	Bladder cancer	1138	924	208	6	1129	908	208	13	Yes	0.10
Matullo [32]		2005	Bladder cancer	156	133	22	1	109	94	13	2	Yes	0.08
Hung [38]		2008	Lung cancer	2417	2126	281	10	3812	3324	470	18	Yes	0.07
Zienolddiny [39]		2005	Lung cancer	312	203	102	7	293	246	45	2	Yes	0.08
Bastos [28]		2009	Thyroid cancer	109	95	14	0	217	181	36	0	Yes	0.08
Fayaz [29]		2012	Thyroid cancer	50	43	7	0	50	45	5	0	Yes	0.05
García-Quispes [14]		2011	Thyroid cancer	397	314	79	4	477	383	90	4	Yes	0.10

* Number °f case-control studies separately reported by articles.

^**^Studies included only in the subgroup meta-analysis of ethnicity.

N: sample size in case or control group; NA: not available; HWE: Hardy–Weinberg equilibrium; MAF: minor allele frequency.

### Quantitative analysis

Given the fact that eligible case-control studies were mainly composed of breast cancer (N = 15) and ovarian cancer (N = 7), which brought about potential bias to the combined analysis; additionally, the considerable inherent heterogeneity among different cancer types and significant statistical heterogeneity we observed (*P_h_*<0.001) indicated that it might not be informative to pool the data of all types of cancer into a single analysis. Therefore we performed meta-analyses respectively in groups of different cancer types, and the results were summarized in Table 3.

**Table 3:** Stratified analysis of the *XRCC2* Arg188His polymorphism on cancer susceptibility.

Variables			Dominant Model		Recessive Model		Additive Model	
	N**	Case/Control	OR(CI)	P/*P_h_*	OR(CI)	P/*P_h_*	OR(CI)	P/*P_h_*
Breast	14	17420/17811	0.94(0.86,1.04)	0.232/0.013	1.03(0.86,1.23)	0.741/0.402	0.94(0.86,1.04)	0.251/0.004
Caucasian*	11	15192/15360	0.93(0.84,1.03)	0.158/0.012	1.02(0.86,0.22)	0.890/0.270	0.93(0.83,1.04)	0.181/0.003
								
Gynecological								
Ovarian	6	3035/5554	**0.83(0.73,0.95)**	0.007/0.407	0.64(0.35,1.15)	0.136/0.601	**0.82(0.72,0.93)**	0.003/0.424
Endometrial	2	447/895	0.95(0.70,1.27)	0.711/0.645	1.20(0.79,1.81)	0.399/0.553	0.98(0.73,1.30)	0.865/0.654
Cervical	1	117/205	**0.43(0.21,0.87)**	0.019/NA	NA	NA	**0.43(0.21,0.87)**	0.019/NA
								
Others								
UADT	3	499/583	**1.51(1.04,2.20)**	0.032/0.370	2.11(0.50,8.19)	0.309/0.508	**1.55(1.07,2.24)**	0.020/0.251
Colorectal	2	1309/1480	1.10(0.90,1.35)	0.354/0.174	1.71(0.80,3.68)	0.169/0.253	1.34(0.74,2.43)	0.332/0.074
Pancreatic	2	410/473	0.98(0.65,1.48)	0.926/0.768	2.17(0.20,24.05)	0.527/NA	1.00(0.67,1.51)	0.991/0.749
Brain	3	528/1404	0.94(0.70,1.26)	0.673/0.529	0.78(0.19,3.22)	0.732/0.804	0.93(0.69,1.24)	0.601/0.560
Thyroid	3	556/744	1.02(0.77,1.36)	0.897/0.515	1.20(0.30,4.84)	0.794/NA	1.02(0.77,1.36)	0.866/0.509
Lung	2	2729/4105	1.59(0.54,4.07)	0.398/<0.001	1.19(0.61,2.30)	0.601/0.135	1.61(0.53,4.86)	0.398/<0.001
Bladder	2	1294/1238	0.96(0.79,1.18)	0.703/0.728	0.44(0.18,1.08)	0.073/0.835	0.93(0.76,1.13)	0.469/0.831
Skin	3	796/2592	0.96(0.77,1.20)	0.709/0.812	1.24(0.55,2.82)	0.605/0.993	0.97(0.78,1.21)	0.789/0.819

*Subgroup analysis.

**Number of studies included.

P: P-value of association test, *P_h_*: P-value of Q-test for heterogeneity test; NA: not available.

### Breast cancer

**Figure 2:**
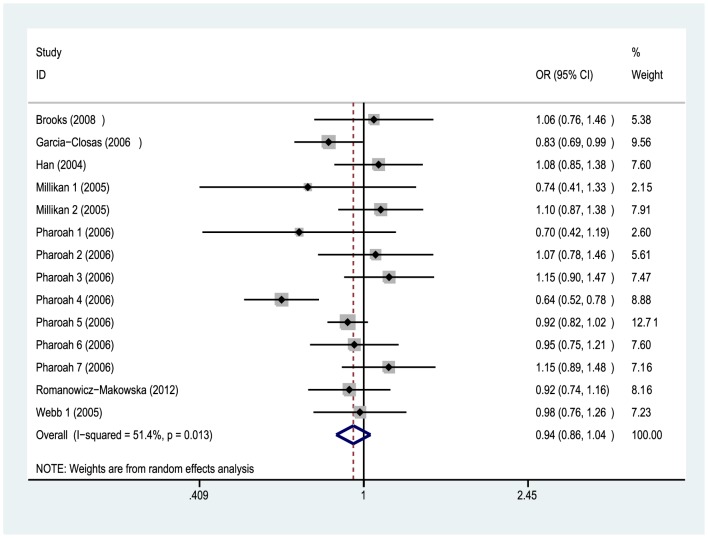
Forest plot for the association of the *XRCC2* Arg188His polymorphism with breast cancer risk (dominant model: His/His + Arg/His vs. Arg/Arg). No significant association was observed between the Arg188His polymorphism and susceptibility of breast cancer.

A total of 17420 breast cancer cases and 17811 controls were included in the meta-analysis. As shown in Table 3, no significant association was observed between the Arg188His polymorphism and breast cancer risk under dominant (OR = 0.94, 95%CI = 0.86–1.04, P = 0.232), recessive (OR = 1.03, 95%CI = 0.86–1.23, P = 0.741, Fig. 2) and additive model (OR = 0.95, 95%CI = 0.87–1.04, P = 0.298). Cumulative meta-analysis obtained no significant association as evidence accumulated by time (data not shown).

**Figure 3:**
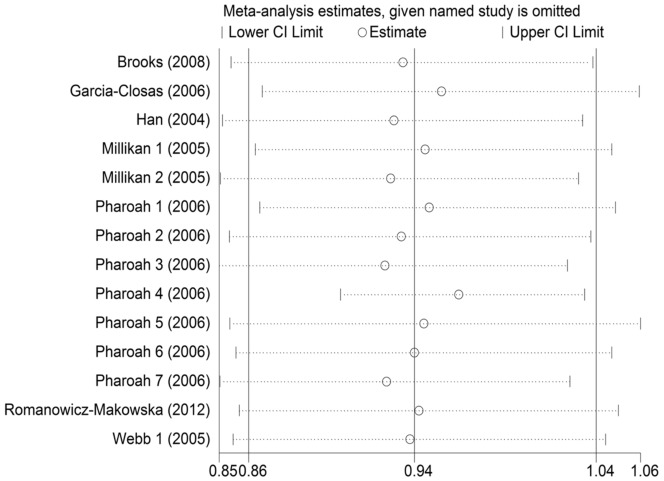
Sensitivity analysis on the association between the *XRCC2* Arg188His polymorphism and susceptibility of breast cancer (dominant model: Arg/His+His/His vs. Arg/Arg). No statistically different results were obtained by excluding every single study in sequence.

There was significant heterogeneity when these breast cancer studies were combined under dominant (*P_h_* = 0.013) and additive model (*P_h_* = 0.005). Hence we carried out stratified analysis by ethnicity,but the heterogeneity did not decrease significantly among Caucasians (Table 3). Results of meta-regression analysis showed that neither ethnicity (Coef. = 1.079, P = 0.171) nor source of controls (Coef. = 1.057, P = 0.432) contributed significantly to the heterogeneity. Sensitivity analysis found that ORs did not change significantly when excluding each single study by sequence and verified the stability of our results to some degree (Fig.3). It was worth mentioning that no significant heterogeneity (Dom: *P_h_* = 0.420, Add: *P_h_* = 0.712) was detected when one case-control study [21](“Pharoah 4” in Table 1) from USA was excluded, which implied that this study might be the origin of the heterogeneity under dominant and additive model.

**Figure 4:**
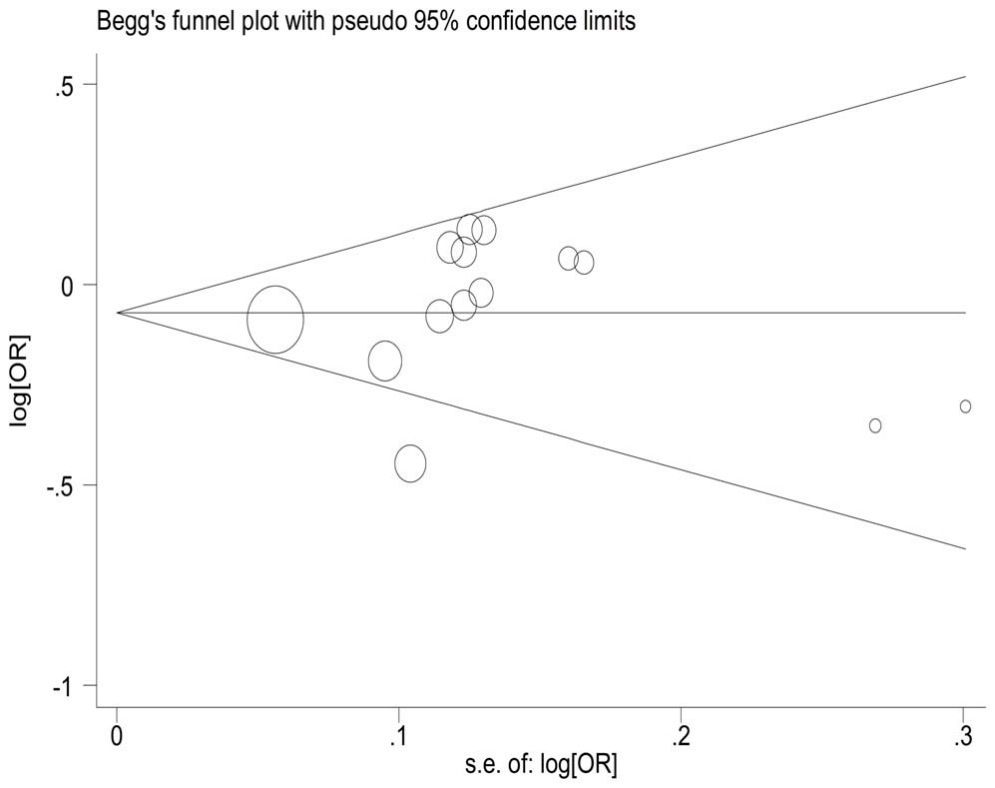
Begg's funnel plot on publication bias for eligible studies that focused on the association of the *XRCC2* Arg188His polymorphism with the breast cancer susceptibility (dominant model: Arg/His+His/His vs. Arg/Arg). The funnel plot seemed symmetrical, indicating no publication bias.

The Begg's funnel plot of dominant model seemed symmetrical (Fig. 4), and Egger's test provided statistical evidence which identified the absence of publication bias (Dom: t = 0.41, p = 0.690). Results of recessive and additive model showed no significant publication bias either (data not shown).

### Gynecological cancer

**Figure 5:**
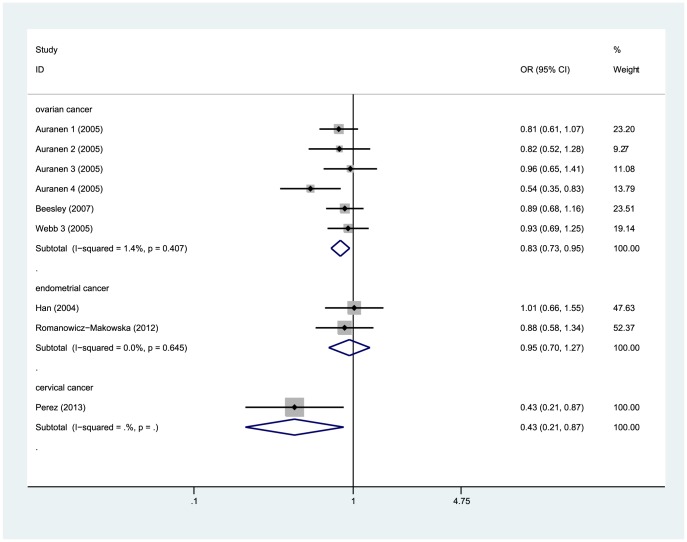
Forest plot for the subgroup analysis of gynecological cancer (dominant model: Arg/His+His/His vs. Arg/Arg). Significant association was detected between the *XRCC2* Arg188His polymorphism and decreased risk for ovarian cancer and cervical cancer.

Considering 6 of the 10 studies investigated ovarian cancer, and it might generate misleading results for the combined analysis of all types of gynecological cancer, we conducted meta-analysis separately for each type of gynecological cancer. We observed that variant allele carriers (His/His + Arg/His) had significantly lower risk for developing ovarian cancer (OR = 0.83, 95%CI = 0.73–0.95, P = 0.007, Fig.5). However, no significant association was detected between this polymorphism and endometrial cancer (Fig.5). Only one study focusing on cervical cancer reported significant association between the Arg188His polymorphism and decreased susceptibility. Moreover, we did not detect any significant heterogeneity among the eligible studies in each comparison (details in Table 3). For ovarian cancer, the Begg's funnel plot and Egger's test indicated no significant publication bias under the three different genetic models (data not shown). The result of sensitivity analysis for ovarian cancer group found the ORs did not change significantly when every single study were excluded.

### Other Cancers

**Figure 6:**
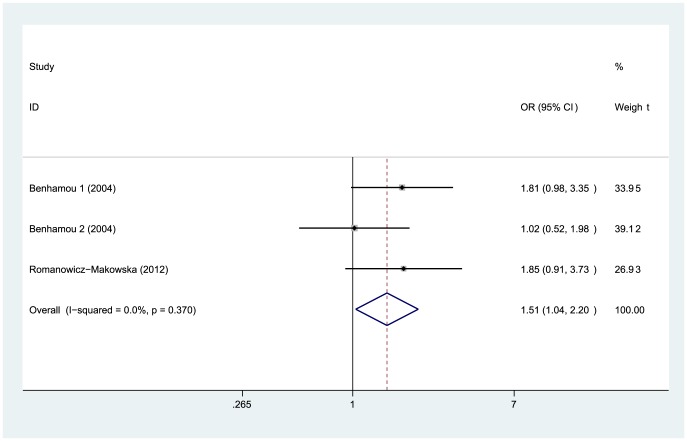
Forest plot for the association between the *XRCC2* Arg188His polymorphism and UADT cancer risk (dominant model: Arg/His+ His/His vs. Arg/Arg). Significant association was observed between this polymorphism and increased risk for UADT cancer.

Relatively small number of studies covered other types of cancers. In our study, the UADT cancer consisted of oral, pharyngeal and laryngeal cancer. Significant association was observed between the Arg188His polymorphism and increased susceptibility of UADT cancer (Dom: OR = 1.51, 95%CI = 1.04–2.20, P = 0.032, Fig.6; Add: OR = 1.51, 95%CI = 1.06–2.16, P = 0.023), and no significant heterogeneity was found in any genetic model (Table 3). As for digestive system cancers, we detected no significant association between this polymorphism and either pancreatic or colorectal cancer. In addition, current evidence did not suggest that the Arg188His polymorphism was associated with risk for brain, skin, thyroid, bladder and lung cancer (details in Table 3).

## Discussion

As research moved along, the genomic landscape of cancer has been gradually brought into light, and an increasing number of vital genes shared by various cancers, *XRCC2* for example, have been revealed in recent years. The *XRCC2* Arg188His polymorphism was widely reported to be associated with susceptibility of a wide range of cancers. However, results remained conflicting and a single study might be limited due to a relatively small sample size. Moreover, no conclusive study so far has reported a result which covered all available cancer types. Based on all published literature, we performed this meta-analysis to identify the association of the Arg188His polymorphism with cancer susceptibility.

Our study, which derived an asymmetrical distribution of different cancer types, contained 15 studies for breast cancer and 7 for ovarian cancer, generating potential bias to the combined analysis of all cancer types. Moreover, considerable inherent heterogeneity existed among different cancer types, which was confirmed by significant statistical heterogeneity we obtained. Current evidence indicated that the *XRCC2* Arg188His polymorphism might play various roles in different cancer types. Thus it could be of little value to combine all data of different cancer types into a single analysis.

According to our meta-analysis of breast cancer, we observed no significant association between this polymorphism and susceptibility of breast cancer, which accorded with the previous meta-analysis. However, in the previous study, Yu et al [10]reported their result with unexplained significant heterogeneity (*Ph* = 0.014). Furthermore, studies inconsistent with HWE were included in the meta-analysis which might result in potential bias. Limited by factors above, results of previous study should be interpreted with caution. In our study, we detected no significant heterogeneity when one case-control(“Pharoah 4” in Table 1) study was [21] excluded, which implied the probability of the removed study being the origin of heterogeneity. We noted that in this case-control study from USA, buccal cells as DNA samples were collected by mail from participants ,which could brought in possible inaccuracy. However, insufficient information was provided for further identification of the heterogenous factor of this study. Additionally, cumulative meta-analysis suggested that no significant association was observed as evidence accumulated by time. Theoretically, genetic variants in the *XRCC2* gene could change the regular function of this gene, disturb the DNA repair and increase cancer risk. However, a previous study [40] has identified that the variant allele of this polymorphism could increase resistance to the DNA damage induced by cisplatin, which enlightened protective function of this polymorphism under certain conditions. This finding is to some degree consistent with our paradoxical result of the non-significant association between this polymorphism and breast cancer risk. Future studies should aim at the response of variant allele carriers to specific DNA damage agents of breast cancer.

As for gynecological cancer, we found that variant allele carriers had significantly lower risk for developing ovarian cancer. Our results suggested a protective role of the Arg188His polymorphism, which was apparently paradoxical to the presumable hypothesis. As mentioned above, the previous study [40] indicated that the Arg188His polymorphism might response differently to various damaging agents. It is notable that in one of the studies included in our meta-analysis, Pérez et al [27] adopted HPV adjusted ORs and found the variant allele (A allele) was associated with reduced risk for cervical cancer, which derived a hypothesis that this polymorphism might play a different role in HPV-induced DNA damage. However, our paradoxical findings of gynecological cancer should be interpreted with caution, because only a small number of studies investigating ovarian, endometrial and cervical cancer were finally included in our meta-analysis. A large number of studies with specific damaging factors in consideration should be accumulated to provide further estimate on the association between this polymorphism and gynecological cancer risk.

In our study, we detected significant association between the Arg188His polymorphism and increased UADT cancer, but the result was limited by the small sample size. Considerable caution should be taken into account because only 3 studies were included. Meanwhile, biological heterogeneity is likely to exist among oral cavity, pharyngeal and laryngeal cancer. A few studies investigated other cancer types (brain, skin, thyroid, pancreatic, colorectal cancer); however, we found no significant results in these subgroups. More studies are required to achieve conclusive results for these cancer types and future work should cover cancers that have not been investigated such as gastric, esophageal and hematological cancer.

To our knowledge, this is the most comprehensive meta-analysis which has first investigated the association between the *XRCC2* Arg188His polymorphism and susceptibility of all available cancer types. However, several limitations should be taken into consideration when explaining the results: (1) we only included the studies from selected databases, thus other relevant records might be left out; (2) the number of studies include was relatively small for some cancer types. For example, only one study investigated cervical cancer; (3) due to insufficient information, stratified analysis cannot be conducted by age, sex, treatment, drinking status, exposure to radiation and other factors;(4) lacking of necessary data limited further assessment for gene-gene and loci-loci interaction; (5) as only Caucasian and African American were involved in the pooled analysis, the results might not suit for other ethnicities.

In conclusion, our meta-analysis found that the impact of the *XRCC2* Arg188His polymorphism on susceptibility of different cancers might be diverse. Current evidence did not suggest this polymorphism was directly associated with breast cancer risk. However, we observed that the variant allele carriers might have significantly lower risk for developing gynecological cancer, especially ovarian cancer. Our results should be explained with some caution and be re-evaluated in the future when more studies with larger sample size are conducted.

## Supporting Information

Checklist S1 PRISMA checklist.
(DOC)
